# Detection of equine herpesvirus-4 and physiological stress patterns in young Thoroughbreds consigned to a South African auction sale

**DOI:** 10.1186/s12917-015-0443-4

**Published:** 2015-06-02

**Authors:** Marcha Badenhorst, Patrick Page, Andre Ganswindt, Peter Laver, Alan Guthrie, Martin Schulman

**Affiliations:** Department of Companion Animal Clinical Studies, Faculty of Veterinary Science, University of Pretoria, Private Bag XO4, Onderstepoort, 0110 South Africa; Department of Anatomy and Physiology, Faculty of Veterinary Science, University of Pretoria, Private Bag XO4, Onderstepoort, 0110 South Africa; Equine Research Centre, Faculty of Veterinary Science, University of Pretoria, Private Bag XO4, Onderstepoort, 0110 South Africa; Section of Reproduction, Department of Production Animal Clinical Studies, Faculty of Veterinary Science, University of Pretoria, Private Bag XO4, Onderstepoort, 0110 South Africa

**Keywords:** Horse, Equine herpesvirus, Physiological stress, Sales consignment, Faecal glucocorticoid metabolites

## Abstract

**Background:**

The prevalence of equine herpesvirus types-1 and -4 (EHV-1 and -4) in South African Thoroughbreds at auction sales is currently undefined. Commingling of young Thoroughbreds from various populations together with physiological stress related to their transport and confinement at a sales complex, may be associated with shedding and transmission of EHV-1 and -4. This prospective cohort study sampled 90 young Thoroughbreds consigned from eight farms, originating from three provinces representative of the South African Thoroughbred breeding demographic to a sales complex. Nasal swabs for quantitative real-time polymerase chain reaction (qPCR) assay to detect EHV-1 and -4 nucleic acid and blood samples for enzyme-linked immunosorbent assay for EHV-1 and -4 antibodies were collected from all horses on arrival and departure. Additional nasal swabs for qPCR were obtained serially from those displaying pyrexia and, or nasal discharge. Daily faecal samples were used for determination of faecal glucocorticoid metabolite (FGM) concentrations as a measurement of physiological stress and these values were modelled to determine the factors best explaining FGM variability.

**Results:**

EHV-4 nucleic acid was detected in 14.4 % and EHV-1 from none of the animals in the study population. Most (93.3 %) and very few (1.1 %) of this population showed antibodies indicating prior exposure to EHV-4 and EHV-1 respectively. Pyrexia and nasal discharge were poor predictors for detecting EHV-4 nucleic acid. The horses’ FGM concentrations increased following arrival before decreasing for most of the remaining study period including the auction process. Model averaging showed that variation in FGM concentrations was best explained by days post-arrival and transport duration.

**Conclusions:**

In this study population, sales consignment was associated with limited detection of EHV-4 nucleic acid in nasal secretions, with most showing prior exposure to EHV-4 and very few to EHV-1. The physiological stress response shown by most reflected the combination of stressors associated with transport and arrival and these are key areas for future investigation into management practices to enhance health and welfare of young Thoroughbreds during sales consignment.

## Background

Equine respiratory infection is an important cause of disease and economic loss worldwide resulting in significant wastage, particularly in the Thoroughbred racing industry, due to lost training days, prolonged absence from racing and possible negative effects on long-term athletic performance [1, 2]. Equine herpesvirus types-1 and -4 (EHV-1 and -4) are important agents associated with infectious upper respiratory tract disease (IURD) [3–5]. Few surveillance studies to detect these viruses are reported in healthy horse populations [6, 7].

Risks associated with IURD are multifactorial, including host, environmental, management and pathogen-specific factors [8, 9]. Age as a risk factor in juvenile horses has been well recognised with higher detection rates of EHV-1 and -4 reported during the colder winter months [3, 4, 8–12]. Physiological stress is arguably one of the more important risk factors, with associations reported between EHV-1 and -4 recrudescence and shedding and various exogenous stressors, as well as experimental corticosteroid administration [13–15]. Stress may further increase the susceptibility of naive animals to new infections [13]. Transport and the subsequent confinement, handling and management at sales events may contribute to physiological stress [10, 12, 13, 16]. Large, intermingled assemblages of horses from diverse sources provide an opportune environment for viral shedding and transmission, exacerbated by potentially stressful disruption of established social groups [9, 10, 17]. Detection of EHV-1 from nasal secretions following long distance transport of horses and both EHV-1 and -4 upon horses’ arrival and shortly thereafter at North American sales and show events have been reported [12, 16].

Measurement of faecal glucocorticoid metabolite (FGM) concentrations to monitor adrenocortical function in horses provides a practical, non-invasive and feedback-free alternative to glucocorticoid (e.g., cortisol) determination in blood, saliva, urine or milk [17, 18]. FGM concentrations unlike rapidly-fluctuating blood cortisol levels reflect cumulative secretion and elimination of hormones over an extended interval. A delayed, time-averaged response to a stressor is provided dependant on species-specific gut-passage times, with an interval of approximately 24 h in horses [18, 19]. Fluctuations in FGM concentrations have been reported for determining stress responses in horses, including animals consigned to sales and following short, medium and long-distance road transport [17, 20, 21].

The effects of sales consignment on detection of viral nucleic acids and physiological stress responses among young Thoroughbreds have not been reported previously. This study aimed to detect EHV-1 and -4 nucleic acids in nasal secretions of young Thoroughbreds at a South African auction sale. It also aimed to determine which factors among: EHV-4 nucleic acid detection, clinical signs of respiratory disease, transport and preparation for auction explained variability in FGM concentrations, as an indicator for physiological stress.

## Methods

The study was approved by the Animal Ethics Committee of the University of Pretoria (Study V040-13).

### Animals

The study population included 90 (51 colts, 39 fillies) of the 358 two-year old Thoroughbred horses catalogued for an annual auction sale in South Africa. The horses enrolled were pre-selected from the sales catalogue based on owners’ consent to participate and the availability of accurate records from the particular farms. Selected horses originated from eight farms situated in three provinces: 30 from three (Farms 1, 2 and 3) in the Western Cape Province, 18 from two (Farms 4 and 5) in the Eastern Cape Province and 42 from three (Farms 6, 7 and 8) in KwaZulu-Natal Province. This selection was representative of the South African Thoroughbred breeding demographic.

Housing at the complex consisted of 44 barn-style buildings, subdivided into 771 individual stables and separated by walkways. Each farm’s consigned horses were allocated a unique stable number and housed in adjacent stables, although often within the same barn as horses from other farms. The buildings’ design allowed for free movement of air between stables and windows with metal grids enabled nose-to-nose contact between horses in adjacent stables. Horses were periodically removed from their stables into adjacent walkways for showing or grooming and mixed randomly at communal facilities during routine daily activities including in-hand exercise and washing. Daily care activities of the horses, including feeding, were performed by the staff of the respective farms according to each farm’s protocol. All enrolled horses were vaccinated against equine influenza virus, African horse sickness and tetanus, but none against EHV-1 or -4. Informed, written consent for participation was obtained from the owners of each participating farm.

### Study design

A prospective cohort study was performed during the late southern hemisphere winter at the Thoroughbred Breeders Association National Two Year Old Sales in Germiston, Gauteng Province of South Africa. Horses travelled by road transport and arrived at the sales complex on various dates. Their residence period therefore varied between four and nine days. The auction occurred over two days (15 and 16 August 2013), with each horse’s date of auction regarded as the end-point of clinical data collection for that horse, prior to its departure. For half of the horses, FGM data were also collected on the day post auction. For each horse, the period from its day of arrival until the third day post-arrival was defined as the ‘adaptation phase’. For each horse, the period including the day prior to and the day of its auction was defined as the ‘auction phase’. These phases were defined *a priori* to allow comparison of data from horses with varying periods of residence.

### Sample collection and analyses

#### Sample collection

##### At arrival

One nasal swab, a blood sample and a faecal sample were collected from each horse within 24 h of arrival. Nasal secretion samples were collected using a 15 cm metal shaft rayon-tipped swab^1^ advanced into either of the horse’s nostrils and gently rotated against the mucous membranes for collection of nasal secretion and epithelial cells. Following collection the swab was replaced in its sterile, dry plastic tube and refrigerated at 4-6 °C until delivery to the Veterinary Genetics Laboratory, University of Pretoria. Laboratory processing of nasal swabs was performed within 48 h of collection. One 8.5 ml BD Vacutainer® SSTTM II Advance Plus serum tube^2^ was filled with blood from each horse by means of jugular venipuncture. Blood samples were refrigerated at 4-6 °C following collection, until delivery to the Immunocontraception Laboratory, University of Pretoria. A faecal sample was collected from each horse’s stable between 06 h00-09 h00 in a 25 ml plastic specimen container, frozen at -20 °C within 2 h of collection and kept frozen until delivery to the Endocrine Research Laboratory, University of Pretoria.

##### Daily monitoring

From arrival until departure horses were monitored twice daily, between 06 h00-09 h00 and 15 h00-18 h00 with rapid digital thermometers (Thermoval®^3^) for pyrexia, defined as a rectal temperature ≥ 38.5 °C. Horses were additionally monitored once daily, between 06 h00-09 h00, for the presence of an obvious nasal discharge.

##### Daily sampling of horses with pyrexia and, or nasal discharge

Subsequent to recording a pyrexia and, or nasal discharge in any horse, serial nasal swabs were collected daily as described until the day of departure.

#### Daily sampling of study population

Faecal samples were collected daily as described.

#### Prior to departure

One nasal swab, a blood sample and a faecal sample were collected as described from each horse following their auction, within 24 h prior to their departure.

#### Laboratory analyses

##### Quantitative real-time polymerase chain reaction (qPCR) for EHV-1 and -4 deoxyribonucleic acid (DNA)

Nasal swabs were agitated for 5 s in 0.5 ml of 0.1 M phosphate buffered saline (pH 7.4) in a 1.5 ml Eppendorf tube. Nucleic acid was extracted from 100 μl of the preparation using MagMax^TM^ Pathogen DNA/RNA kit^4^ and a Kingfisher 96 Magnetic Particle Processor^5^ according to manufacturer’s protocols. Subsequently, a duplex qPCR for EHV-1 and -4 was performed using previously described primers and probes [22]. Briefly, 17 μl of a master mix consisting of 1 μl of each primer/probe mix, 5 μl of nuclease-free water and 10 μl of Kapa Probe Fast ABI Prism® 2X PCR master mix^6^ was added to each well of a PCR plate and 3 μl of the extracted template was added. EHV-1 and EHV-4 reference viral cultures were obtained from the Equine Virology Research Laboratory, University of Pretoria. Aliquots of nucleic acid extracted from these reference virus cultures were included on each plate as positive controls with nuclease free water being included as a negative control. The qPCR was performed according to the manufacturer’s protocol with the assignment of a cut-off value of < 40 cycles (Ct) for positive detection of viral DNA.

##### Enzyme-linked immunosorbent assay (ELISA) for EHV-1 and -4 antibodies

Each serum sample was tested against the glutathione-S-transferase (GST) fusion proteins of EHV-1 glycoprotein G, EHV-4 glycoprotein G and against GST only, as previously described by Crabb and Studdert [23] and validated by Gilkerson *et al.* [24]. To increase the sensitivity of the assay and assess inter-sample variation, all samples were tested against each antigen in duplicate and the mean absorbance of the two tests was used as the test result. The following cut-off levels were used for interpretation of the absorbance values: > 0.2 for antibody-positive; 0.1-0.2 for indeterminate; and < 0.1 for antibody-negative. Positive control samples for EHV-1 and EHV-4 antibodies were included on each plate. All antigens and positive control samples used in these assays were obtained from the Centre for Equine Virology, University of Melbourne.

##### Faecal extraction and hormone analysis

Frozen faecal samples were lyophilized, pulverized and sifted using a metal mesh strainer to remove fibrous material [25]. Between 0.10-0.11 g of the faecal powder was then extracted by vortexing for 15 min with 80 % ethanol in water (3 ml). Following centrifugation for 10 min at 1500 g, supernatants were transferred into micro-centrifuge tubes and stored at −20 °C until analysis. Extracts were measured for immunoreactive FGM concentrations using an enzyme immunoassay that detects 11,17-dioxoandrostanes, previously shown to provide reliable information on adrenocortical function in various mammals, including horses [26–28]. Serial dilutions of extracts gave displacement curves parallel to the standard curve of the assay. Sensitivity of the assay at 90 % binding was 1.8 ng/g faeces. Intra- and inter-assay coefficients of variation, determined by repeated measurement of high- and low-value quality controls, ranged between 1.9 % and 16.5 %. The assay was performed as previously described using antibodies for which cross-reactivity’s have been reported [29, 30].

#### Statistical analyses

To determine which factors best explained variability in physiological stress in our study animals, we modelled the natural-log-transformed FGM concentrations from 655 faecal samples in linear mixed models, fitted with the ‘identity’ link function, using *lmer* in Package ‘lme4’ in R [31]. The global model included six standardized fixed effects: days post arrival; auction phase (including the preparation day one day prior to auction and the auction day); transport duration; pyrexia; nasal discharge; EHV-4 DNA detection. Transport duration ranged from 6 to 22 h (median: 15.25 h). Repeated measures were modelled as random effects: horse identity (1|horse); farm identity (1|farm). Candidate models were evaluated with Akaike’s Information Criterion (AIC_c_) [32]. Model averaging was performed using Akaike weights (*w*_*i*_) of all candidate models [33]. Goodness of fit of parameter estimates was assessed using 85 % confidence intervals and *Ω*_0_^2^ for the global model [34, 35]. Collinearity among covariates was assessed with variance inflation factors (with *a priori* values of > 5 deemed questionable and > 10 deemed unacceptable correlation).

## Results

### EHV nucleic acid detection

No EHV-1 DNA was detected, however EHV-4 DNA was detected in nasal secretions of 13/90 (14.4 %) horses originating from 7/8 participating farms (Tables 1 and 2). A total of 21 swabs positive for EHV-4 DNA were obtained from these 13 horses. Repeated incidents of EHV-4 DNA detection occurred in 4/13 (30.8 %) horses. Both the longest period of continuous detection and the longest interval between consecutive detection events in an individual horse, were four days. Nasal swabs from 1/90 (1.1 %) and 7/90 (7.8 %) horses were positive for EHV-4 DNA on day of arrival and departure, respectively. Details of the temporal pattern of EHV-4 nucleic acid detection during the observation period are shown in Table 1. The EHV-4 qPCR-results for 13 horses from which EHV-4 DNA was obtained in their nasal secretions, are shown in Table 2.

**Table 1:** Proportion of EHV-4 nucleic acid qPCR-positive nasal swabs obtained from 90 Thoroughbreds at an auction sale

Farm of consignment	Province of origin	Consigned horses (n)	Sample dates (August 2013)								
			8	9	10	11	12	13	14	15^a^	16^a^
1	Western Cape	13		0/13	1/8	0/9	0/9	0/9	0/9	0/12	0/5
2	Western Cape	9		0/9	2/5	0/5	0/5	0/6	1/7	1/8	1/5
3	Western Cape	8	0/8	0/5	0/5	0/5	1/6	1/7	1/7	1/8	1/5
4	Eastern Cape	13			1/13	0/4	0/4	0/7	0/7	3/12	0/4
5	Eastern Cape	5			0/5	0/1	0/3	1/3	2/4	1/5	0/1
6	KwaZulu-Natal	10				0/10	0/0	1/3	0/5	0/8	0/3
7	KwaZulu-Natal	26		0/26	0/16	0/16	0/17	0/18	0/19	1/25	0/9
8	KwaZulu-Natal	6						0/6	0/1	0/6	0/2
Number (%)		90	0/8 (0)	0/53 (0)	4/52 (7.7)	0/50 (0)	1/44 (2.3)	3/59 (5.1)	4/59 (6.8)	7/84 (8.3)	2/34 (5.9)

^a^ = auction dates

**Table 2:** qPCR-results from 13 Thoroughbreds with detection of EHV-4 nucleic acids in their nasal secretions

= horse not present at sales complex; (−) = EHV-4 qPCR-negative; (^a^) = EHV-4 qPCR-positive with Ct (cycle threshold) value; n/s = no swab collected; ∞ = sampling not possible

### EHV-4 nucleic acid detection and clinical signs

All 13 horses from which nasal swabs positive for EHV-4 DNA were obtained showed either nasal discharge alone, or both pyrexia and nasal discharge during the observation period (Table 3). However, 65/77 (84.4 %) horses in which EHV-4 DNA was not detected also showed one or both of these clinical signs (Table 3). Duration of pyrexia was less than 24 h in 7/8 horses with concurrent detection of EHV-4 DNA.

**Table 3:** Detection of EHV-4 nucleic acid and clinical signs recorded for 90 Thoroughbreds at an auction sale

EHV-4 nucleic acid	Pyrexia	Nasal discharge	Number (%) of study population
-	-	-	12 (13.3)
-	+	-	13 (14.4)
-	-	+	25 (27.8)
-	+	+	27 (30)
+	-	-	0 (0)
+	+	-	0 (0)
+	-	+	5 (5.6)
+	+	+	8 (8.9)

(+) = detected; (−) = not detected

### EHV-1 and -4 serology

Upon arrival at the sales complex, 1.1 % and 93.3 % of the study population showed serological evidence of prior exposure to EHV-1 and EHV-4, respectively. No instances of seroconversion were recorded between arrival and departure. Only one horse (Horse E) of the 13 from which qPCR-evidence of EHV-4 DNA was detected in its nasal secretions, was EHV-4-seronegative on arrival and remained seronegative on departure seven days later.

### FGM concentrations

**Fig. 1:**
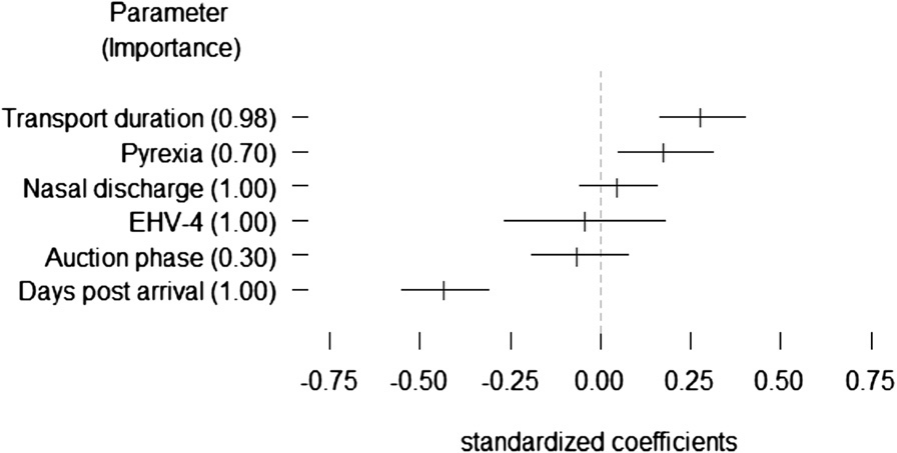
Model-averaged standardized parameter estimates with 85 % confidence intervals for covariates explaining faecal glucocorticoid metabolite (FGM) concentrations in horses. Importance indicates the sum of Akaike weights for all models containing the parameter

None of the covariates in our models exhibited unacceptable collinearity: all variance inflation factors were < 2.2. Several models contained a similar amount of information or explained a similar amount of variability in FGM’s (i.e., ‘best models’ with AICc < 10, Table 4), suggesting that model averaging was an appropriate approach. Based on model averaging, the covariates that best explained variation in FGM concentrations were days post arrival, transport duration, and pyrexia, which all had large standardized effect sizes and differed from zero (Fig. 1). Days post arrival was selected in all of the best candidate models, but transport duration was almost as important (Fig. 1) and was only left out of two of the best models (Table 4). Although the parameter estimate for pyrexia differed from zero, this covariate had a relatively smaller standardized effect size than those of the transport-associated parameters (Fig. 1) and it had relatively lower importance (it was not selected in the second best candidate model, Table 4). The auction phase, EHV-4 DNA detection, and nasal discharge parameters all had either high variability in parameter estimates or a small effect size (Fig. 1). The global model explained 31 % of variation in FGM concentrations, with *Ω*_0_^2^ = 0.31.

**Table 4:** Models with Akaike weights (w_i_) > 0 and ΔAICc < 10, modelling faecal glucocorticoid metabolite concentrations in horses

Model: log(FGM) ~	log*L*	*K*	AICc	Δ	*w*_*i*_
days + duration + discharge + EHV-4 + pyrexia + (1|farm) + (1|horse)	−364.6	9	747.8	0.0	0.48
days + duration + discharge + EHV-4 + (1|farm) + (1|horse)	−366.5	8	749.5	1.7	0.21
days + duration + discharge + EHV-4 + pyrexia + auction + (1|farm) + (1|horse)	−364.4	10	749.5	1.7	0.21
days + duration + discharge + EHV-4 + auction + (1|farm) + (1|horse)	−366.4	9	751.2	3.5	0.08
days + discharge + EHV-4 + pyrexia + (1|farm) + (1|horse)	−369.6	8	755.5	7.7	0.01
days + discharge + EHV-4 + pyrexia + auction + (1|farm) + (1|horse)	−369.2	9	756.9	9.1	0.01

Individual horses (1|horse) and farms (1|farm) were random effects in all models. Fixed effects were days post arrival (days), auction phase (auction), duration of transport (duration), pyrexia, nasal discharge (discharge), and EHV-4 DNA detection

FGM, faecal glucocorticoid metabolites; EHV-4, equine herpesvirus-4

**Fig. 2:**
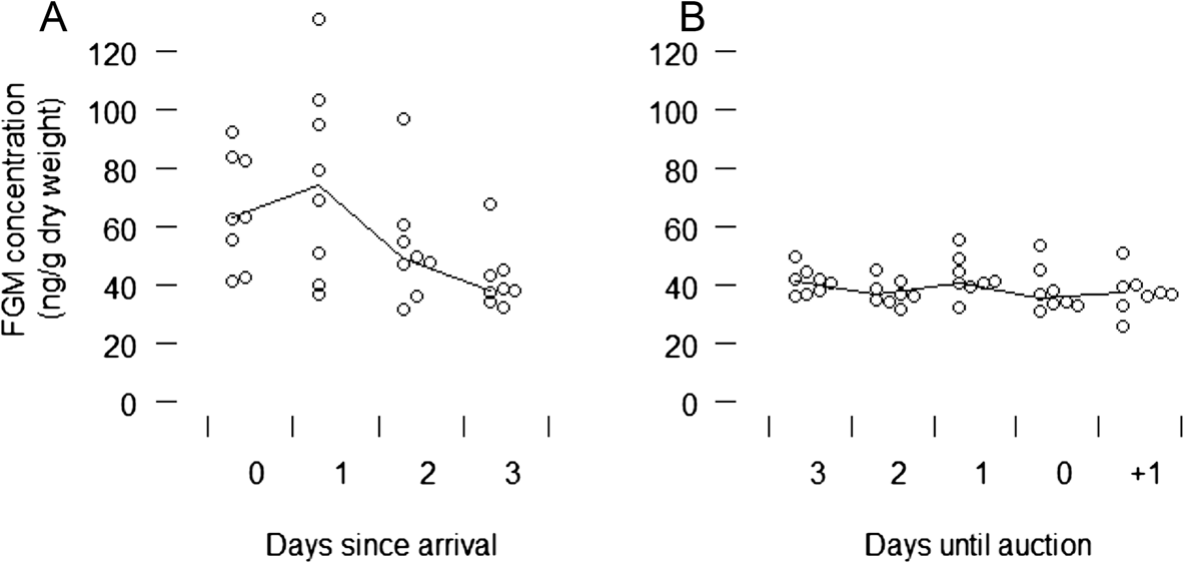
Faecal glucocorticoid metabolite (FGM) concentrations of consigning farms (n = 8) during the adaptation (**A**) and auction (**B**) phase. Circles represent median FGM concentrations for horses from different farms on each day. Lines connect daily medians of the 8 farm median FGM values. Day “+1” in panel B indicates the day post-auction for which faecal samples were obtained from half of the study’s horses. With a gut passage time of approximately 24 h, FGM values reflect physiological stress responses to stimuli experienced on the previous day

*Post hoc* graphical analysis of the FGM data supported the results of the model averaging and suggested that median FGM concentrations for the eight farms increased (with increased variability) after arrival, before decreasing in concentration and variability (to approximately 40 ng/g dry weight) for most of the remainder of the study period (Fig. 2). During the adaptation phase, FGM concentrations were 64 % higher and 93 % higher on the day of arrival and one day after arrival, respectively, when compared to three days after arrival (Fig. 2A, and represented by the largest effect size in Fig. 1). No discernible increase in FGM concentrations was associated with the auction phase (Fig. 2B, and represented by small effect size with a confidence interval overlapping zero in Fig. 1).

## Discussion

Detection rates of EHV-4 and EHV-1 DNA in nasal secretions from the study population horses were higher and lower, respectively, than those reported in populations showing clinical signs of respiratory disease [4, 5]. It was previously suggested that EHV-1 rarely circulates outside breeding populations inclusive of young foals [12]. In the present study, EHV-4 DNA detection included single and repeated, continuous or interrupted events and the relatively low rate of detection upon sales arrival was similar to a previous report [12]. Most EHV-4 detection events coincided with the dates of the two auction days, peaking on the first of these days. Both viral recrudescence and horizontal spread of primary infection may have contributed to the increased detection of EHV-4 DNA observed between arrival and departure [13, 14]. The study population showed a greater seroprevalence of EHV-4 compared to EHV-1, reflecting that prior exposure on the farms of origin was almost universal or only in a few individuals, to EHV-4 and EHV-1 respectively. This seroprevalence was similar to previous reports from different populations, including two-year old racehorses in Australia [13, 36, 37]. EHV-4 DNA was detected in the nasal secretions of 12 horses despite the presence of EHV-4 antibodies, most likely as a result of viral recrudescence. In the case of Horse E, EHV-4 DNA detection likely resulted from recent primary EHV-4 infection with nasal shedding of virus and serum sample collection prior to establishment of a detectable antibody response. The implications of EHV-1 and -4 DNA detection at sales departure and the associated risks of viral shedding and transmission at subsequent destinations, including training facilities, warrant further investigation.

In this study we observed a similar prevalence of pyrexia and a higher prevalence of nasal discharge than previously reported among EHV-4-positive horses [4]. Clinical signs was a poor indicator for viral nucleic acid detection, with several horses that showed clinical signs being negative for EHV-4 DNA, contrasting with a reported association between clinical signs of respiratory disease and EHV-4 detection in foals [38]. A pyrexia duration < 24 h in the majority of EHV-4 qPCR-positive horses supported the utility of twice daily rectal temperature monitoring for suspected clinical cases [9, 39]. A study limitation was the reliance of nasal swabbing on observation of clinical signs, potentially resulting in lower detection rates of sub-clinical EHV-1 and -4 infections. The combination of pyrexia and nasal discharge was reported in association with molecular evidence of lesser characterised respiratory viruses EHV-2, EHV-5, equine adenovirus-1 and equine rhinitis B [40]. The current study’s discrepancy in the prevalence of clinical signs and detection of EHV-1 and -4 warrants further investigation of the association of IURD with alternative infectious agents during sales consignment.

Sales consignment was associated with an elevation in FGM concentrations shortly after arrival. This presumably reflected a cumulative series of stressful events associated with transport and sales arrival, and gradually decreased as horses became accustomed to environmental and routine changes. The covariates that best explained the variation in FGM concentration in order of importance were the number of days post-arrival, transport duration and pyrexia. The EHV-4 DNA detection threshold on qPCR was lower than that reported by Diallo *et al.* [22] which may explain the observation that EHV-4 DNA was not an important covariant in our model. Notably, the auction process itself did not appear to initiate any prolonged physiological stress.

Practicalities precluded monitoring of case-matched horses on the farms of origin for EHV-1 and -4 DNA detection and FGM alterations.

## Conclusions

EHV-4 DNA was detected in nasal secretions of some young Thoroughbreds consigned to a South African auction sale. Most of these horses had been exposed to EHV-4 and very few to EHV-1 prior to their arrival at the sale. The combination of stressors associated with their transport and arrival was associated with most horses showing a physiological stress response. These, other stressors and commingling inherent to the current worldwide consignment process increase the risk association with IURD in young horses. The transport and arrival phases are key areas for future investigation into management practices to reduce the impact of physiological stress on the health and welfare of young Thoroughbreds during sales consignment.

## Abbreviations

AIC: Akaike’s information criterion
Ct: Cycle threshold
DNA: Deoxyribose-nucleic acid
EHV: Equine herpesvirus
EHV−1, −2, −4.−5: Equine herpesvirus types−1,−2,−4,−5
ELISA: Enzyme-linked immunosorbent assay
FGM: Faecal glucocorticoid metabolites
GST: Glutathione-S-transferase
PCR: Polymerase chain reaction
IURD: Infectious upper respiratory tract disease
qPCR: Quantitative real-time polymerase chain reaction
RNA: Ribose-nucleic acid

## Footnotes

1 Copan Diagnostics Inc., Murrieta, California, United States of America

2 BD (Becton, Dickinson and Company), Plymouth, United Kingdom

3 Paul Hartmann AG, Heidenheim, Germany

4 Life Technologies, Carlsbad, California, United States of America

5 Thermo Fisher Scientific Inc., Waltham, Massachusetts, United States of America

6 Kapa Biosystems, Cape Town, South Africa
